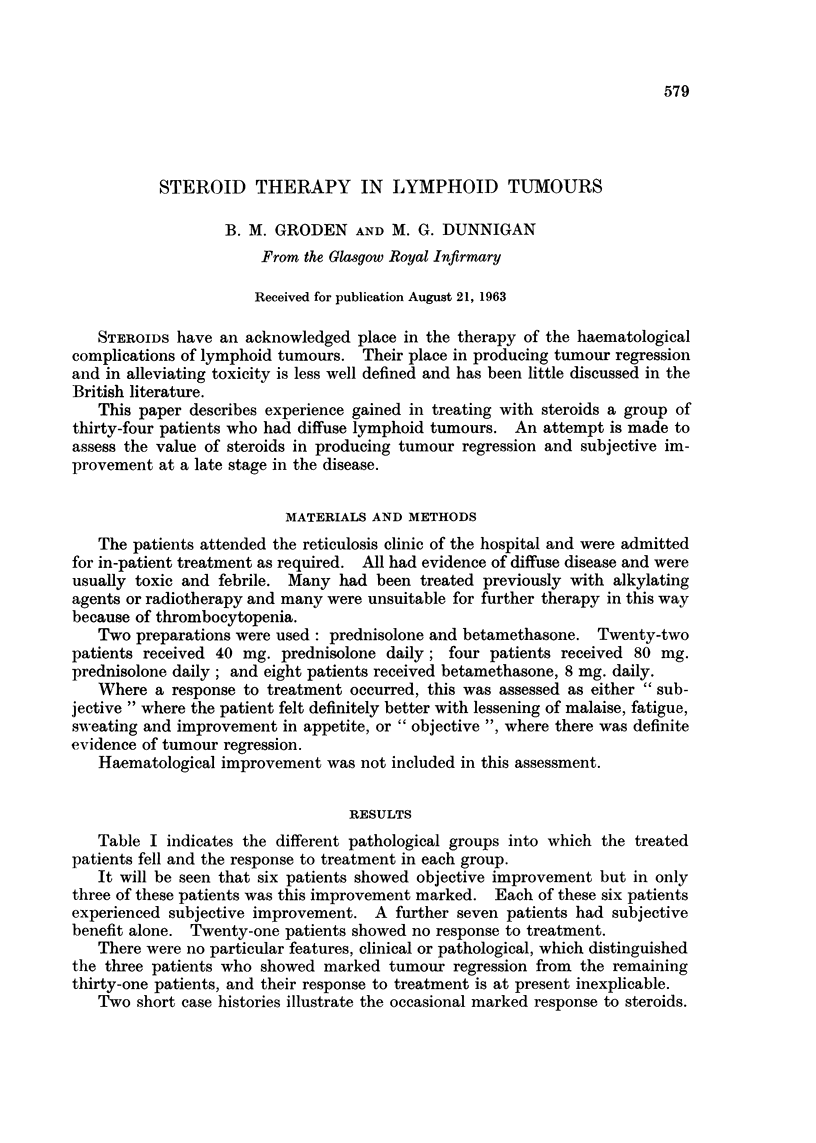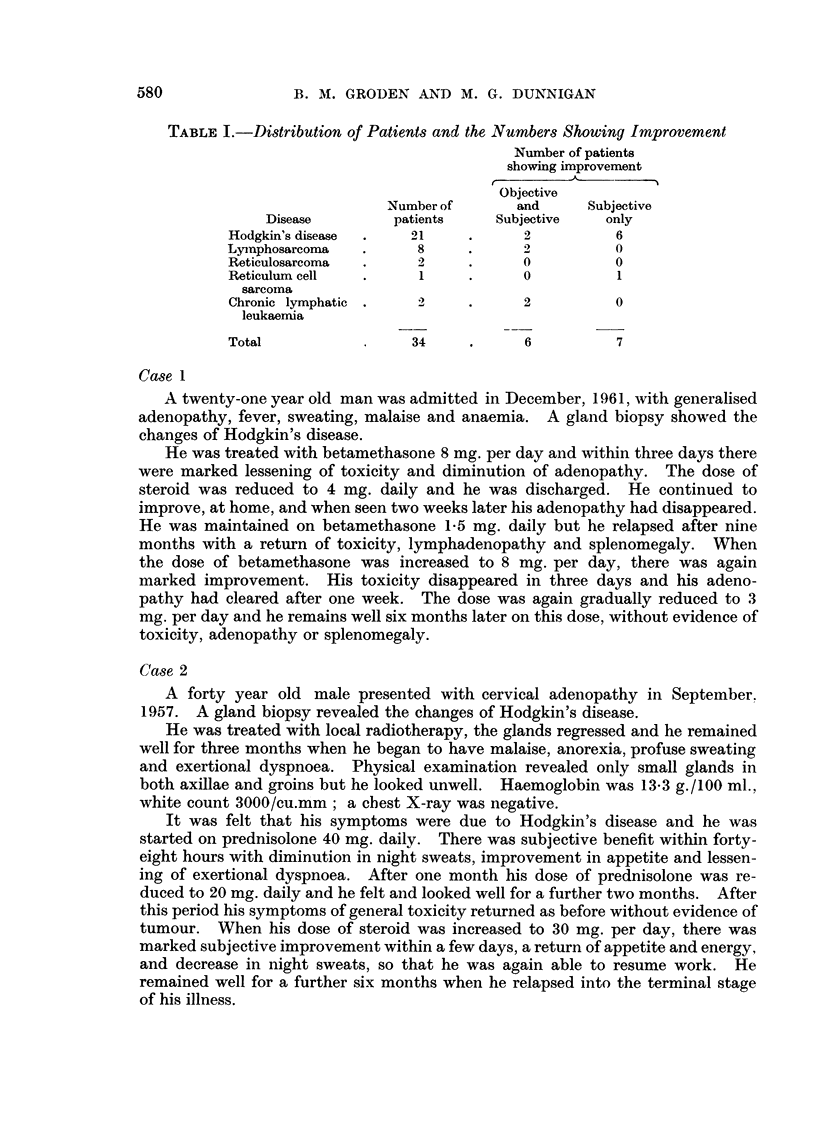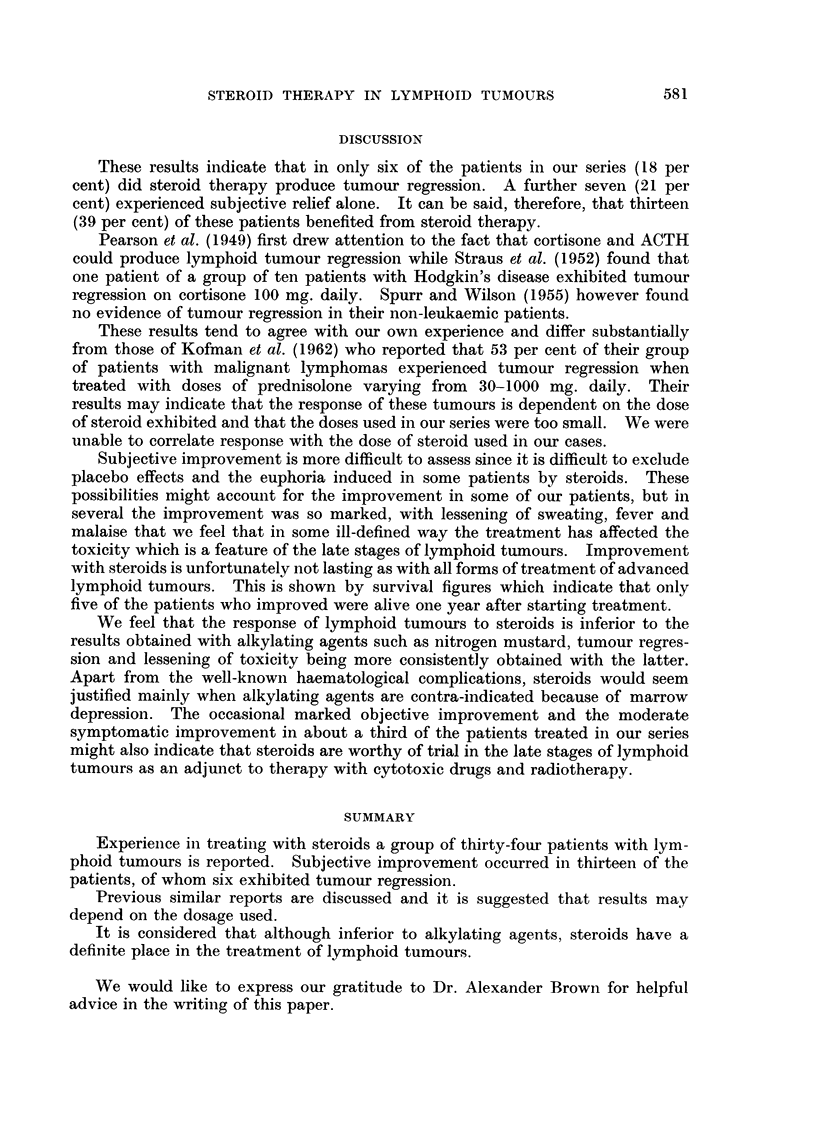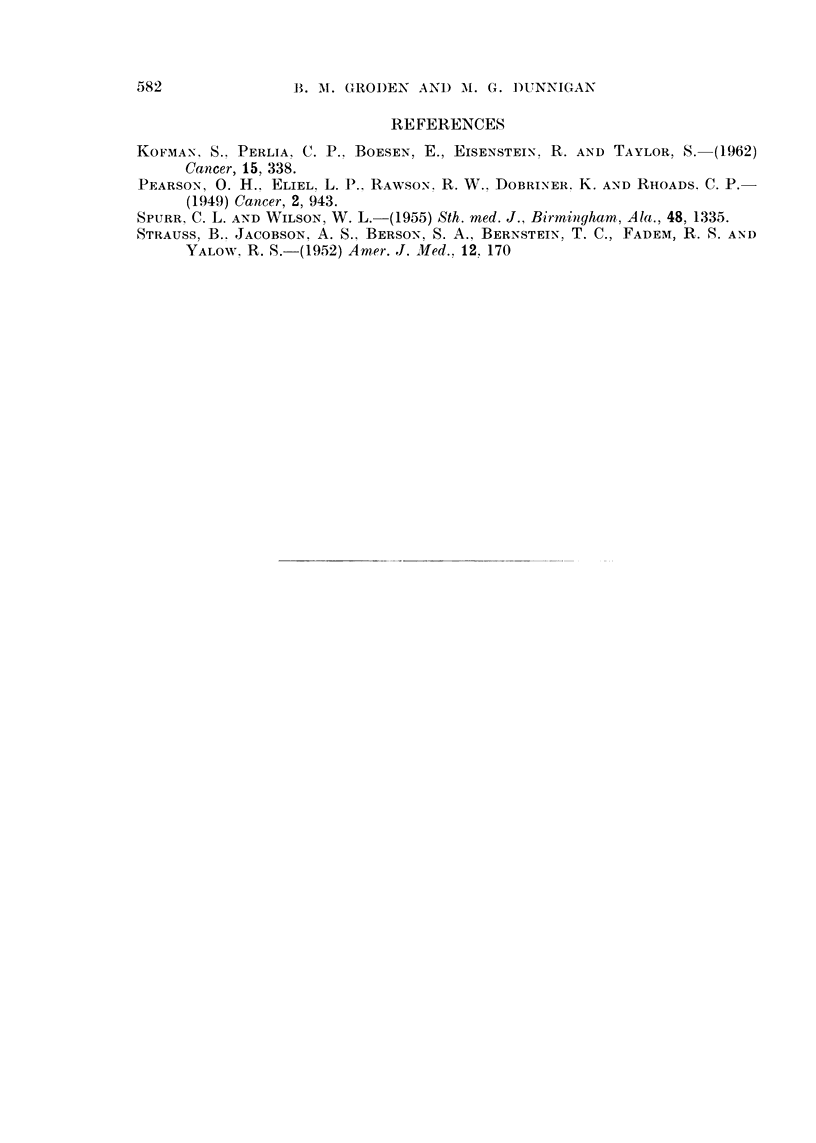# Steroid Therapy in Lymphoid Tumours

**DOI:** 10.1038/bjc.1963.77

**Published:** 1963-12

**Authors:** B. M. Groden, M. G. Dunnigan


### STEROID THERAPY IN LYMPHOID TUMOURS

B. M. GRODEN AND M. G. DUNNIGAN

*From the Glasgow Royal Infirmary*

Received for publication August 21, 1963

STEROIDS have an acknowledged place in the therapy of the haematological complications of lymphoid tumours. Their place in producing tumour regression and in alleviating toxicity is less well defined and has been little discussed in the British literature.

This paper describes experience gained in treating with steroids a group of thirty-four patients who had diffuse lymphoid tumours. An attempt is made to assess the value of steroids in producing tumour regression and subjective improvement at a late stage in the disease.

### MATERIALS AND METHODS

The patients attended the reticulosis clinic of the hospital and were admitted for in-patient treatment as required. All had evidence of diffuse disease and were usually toxic and febrile. Many had been treated previously with alkylating agents or radiotherapy and many were unsuitable for further therapy in this way because of thrombocytopenia.

Two preparations were used : prednisolone and betamethasone. Twenty-two patients received 40 mg. prednisolone daily ; four patients received 80 mg. prednisolone daily ; and eight patients received betamethasone, 8 mg. daily.

Where a response to treatment occurred, this was assessed as either " subjective " where the patient felt definitely better with lessening of malaise, fatigue, sweating and improvement in appetite, or " objective ", where there was definite evidence of tumour regression.

Haematological improvement was not included in this assessment.

### RESULTS

Table I indicates the different pathological groups into which the treated patients fell and the response to treatment in each group.

It will be seen that six patients showed objective improvement but in only three of these patients was this improvement marked. Each of these six patients experienced subjective improvement. A further seven patients had subjective benefit alone. Twenty-one patients showed no response to treatment.

There were no particular features, clinical or pathological, which distinguished the three patients who showed marked tumour regression from the remaining thirty-one patients, and their response to treatment is at present inexplicable.

Two short case histories illustrate the occasional marked response to steroids.

TABLE I.—*Distribution of Patients and the Numbers Showing Improvement*

| Disease | Number of patients | Number of patients showing improvement: Objective and Subjective | Subjective only |
|---|---|---|---|
| Hodgkin's disease | 21 | 2 | 6 |
| Lymphosarcoma | 8 | 2 | 0 |
| Reticulosarcoma | 2 | 0 | 0 |
| Reticulum cell sarcoma | 1 | 0 | 1 |
| Chronic lymphatic leukaemia | 2 | 2 | 0 |
| Total | 34 | 6 | 7 |

*Case* 1

A twenty-one year old man was admitted in December, 1961, with generalised adenopathy, fever, sweating, malaise and anaemia. A gland biopsy showed the changes of Hodgkin's disease.

He was treated with betamethasone 8 mg. per day and within three days there were marked lessening of toxicity and diminution of adenopathy. The dose of steroid was reduced to 4 mg. daily and he was discharged. He continued to improve, at home, and when seen two weeks later his adenopathy had disappeared. He was maintained on betamethasone 1·5 mg. daily but he relapsed after nine months with a return of toxicity, lymphadenopathy and splenomegaly. When the dose of betamethasone was increased to 8 mg. per day, there was again marked improvement. His toxicity disappeared in three days and his adenopathy had cleared after one week. The dose was again gradually reduced to 3 mg. per day and he remains well six months later on this dose, without evidence of toxicity, adenopathy or splenomegaly.

*Case* 2

A forty year old male presented with cervical adenopathy in September, 1957. A gland biopsy revealed the changes of Hodgkin's disease.

He was treated with local radiotherapy, the glands regressed and he remained well for three months when he began to have malaise, anorexia, profuse sweating and exertional dyspnoea. Physical examination revealed only small glands in both axillae and groins but he looked unwell. Haemoglobin was 13·3 g./100 ml., white count 3000/cu.mm ; a chest X-ray was negative.

It was felt that his symptoms were due to Hodgkin's disease and he was started on prednisolone 40 mg. daily. There was subjective benefit within forty-eight hours with diminution in night sweats, improvement in appetite and lessening of exertional dyspnoea. After one month his dose of prednisolone was reduced to 20 mg. daily and he felt and looked well for a further two months. After this period his symptoms of general toxicity returned as before without evidence of tumour. When his dose of steroid was increased to 30 mg. per day, there was marked subjective improvement within a few days, a return of appetite and energy, and decrease in night sweats, so that he was again able to resume work. He remained well for a further six months when he relapsed into the terminal stage of his illness.

### DISCUSSION

These results indicate that in only six of the patients in our series (18 per cent) did steroid therapy produce tumour regression. A further seven (21 per cent) experienced subjective relief alone. It can be said, therefore, that thirteen (39 per cent) of these patients benefited from steroid therapy.

Pearson *et al.* (1949) first drew attention to the fact that cortisone and ACTH could produce lymphoid tumour regression while Straus *et al.* (1952) found that one patient of a group of ten patients with Hodgkin's disease exhibited tumour regression on cortisone 100 mg. daily. Spurr and Wilson (1955) however found no evidence of tumour regression in their non-leukaemic patients.

These results tend to agree with our own experience and differ substantially from those of Kofman *et al.* (1962) who reported that 53 per cent of their group of patients with malignant lymphomas experienced tumour regression when treated with doses of prednisolone varying from 30–1000 mg. daily. Their results may indicate that the response of these tumours is dependent on the dose of steroid exhibited and that the doses used in our series were too small. We were unable to correlate response with the dose of steroid used in our cases.

Subjective improvement is more difficult to assess since it is difficult to exclude placebo effects and the euphoria induced in some patients by steroids. These possibilities might account for the improvement in some of our patients, but in several the improvement was so marked, with lessening of sweating, fever and malaise that we feel that in some ill-defined way the treatment has affected the toxicity which is a feature of the late stages of lymphoid tumours. Improvement with steroids is unfortunately not lasting as with all forms of treatment of advanced lymphoid tumours. This is shown by survival figures which indicate that only five of the patients who improved were alive one year after starting treatment.

We feel that the response of lymphoid tumours to steroids is inferior to the results obtained with alkylating agents such as nitrogen mustard, tumour regression and lessening of toxicity being more consistently obtained with the latter. Apart from the well-known haematological complications, steroids would seem justified mainly when alkylating agents are contra-indicated because of marrow depression. The occasional marked objective improvement and the moderate symptomatic improvement in about a third of the patients treated in our series might also indicate that steroids are worthy of trial in the late stages of lymphoid tumours as an adjunct to therapy with cytotoxic drugs and radiotherapy.

### SUMMARY

Experience in treating with steroids a group of thirty-four patients with lymphoid tumours is reported. Subjective improvement occurred in thirteen of the patients, of whom six exhibited tumour regression.

Previous similar reports are discussed and it is suggested that results may depend on the dosage used.

It is considered that although inferior to alkylating agents, steroids have a definite place in the treatment of lymphoid tumours.

We would like to express our gratitude to Dr. Alexander Brown for helpful advice in the writing of this paper.

### REFERENCES

KOFMAN, S., PERLIA, C. P., BOESEN, E., EISENSTEIN, R. AND TAYLOR, S.—(1962) *Cancer*, **15**, 338.

PEARSON, O. H., ELIEL, L. P., RAWSON, R. W., DOBRINER, K. AND RHOADS, C. P.—(1949) *Cancer*, **2**, 943.

SPURR, C. L. AND WILSON, W. L.—(1955) *Sth. med. J., Birmingham, Ala.*, **48**, 1335.

STRAUSS, B., JACOBSON, A. S., BERSON, S. A., BERNSTEIN, T. C., FADEM, R. S. AND YALOW, R. S.—(1952) *Amer. J. Med.*, **12**, 170